# Symptom burden among men treated for castration-resistant prostate cancer: a longitudinal study

**DOI:** 10.1136/spcare-2024-005054

**Published:** 2024-08-08

**Authors:** Ulrika Rönningås, Per Fransson, Maja Holm, Lars Beckman, Agneta Wennman-Larsen

**Affiliations:** 1 Department of Nursing, Umeå University, Umea, Sweden; 2 Department of Nursing Sciences, Sophiahemmet University, Stockholm, Sweden; 3 Department of Health Care Sciences, Marie Cederschiold hogskola - Campus Ersta, Stockholm, Sweden; 4 Department of Radiation Sciences, Umea Universitet, Umea, Sweden; 5 Department of Clinical Neuroscience, Karolinska Institutet, Stockholm, Sweden

**Keywords:** Prostate, Symptoms and symptom management, Quality of life, Palliative Care

## Abstract

**Objectives:**

Despite rapid expansion of treatments for metastatic castration-resistant prostate cancer (mCRPC) and the importance of symptom management for enhancing quality of life, few studies have focused on men’s experiences of symptom burden over time when receiving one or more lines of treatment in a real-world situation in this phase. The aim was to investigate changes in the multidimensional symptom burden during the first year of life-prolonging treatment of mCRPC.

**Methods:**

Longitudinal data from the first year of life-prolonging treatment for 134 men with mCRPC were used. Symptoms were measured with the multidimensional Memorial Symptom Assessment Scale. Data are presented with descriptive statistics, and changes in symptom burden (physical, psychological and number of symptoms) were analysed using linear mixed modelling.

**Results:**

On average, the men had approximately 10 (0–31) symptoms at inclusion and 12 (0–33) at the last time point. Lack of energy and sweats were the two most reported symptoms at every time point. Sexual problems had the highest scores in all dimensions (frequency, severity, distress). Regarding pain, the distress score was higher than the scores for frequency and severity at t1–t4. Physical symptom burden and the number of symptoms changed significantly over time, towards a higher symptom burden. Psychological symptom burden did not change significantly over time.

**Conclusion:**

The different dimensions of physical symptoms in men treated for mCRPC need to be more acknowledged. Early integration of a palliative care approach could possibly help in enhancing symptom management and quality of life for these men.

**WHAT IS ALREADY KNOWN ON THIS TOPIC**

- An early integration of palliative care is recommended.
- Symptom management is not optimal.

**WHAT THIS STUDY ADDS**

- An understanding of the different dimensions of symptoms.
- An understanding of changes in symptom burden.

**HOW THIS STUDY MIGHT AFFECT RESEARCH, PRACTICE OR POLICY**

- May contribute to effective symptom management.
- May help ensure patients best possible quality of life.

## Introduction

 Prostate cancer is one of the most common cancers in the world,^1^ and until 2004, no evidence-based medical treatments were available when the disease had progressed to a castration-resistant stage. In the past decade, several treatment options offering prolonged survival have been approved^2^,^6^ and men with metastatic castration resistant prostate cancer (mCRPC) may undergo several lines of treatment. In a Swedish cohort consisting of unselected mCRPC patients, median survival from the onset of mCRPC was 13.2–23.2 months depending on whether having metastases already at primary diagnosis or not.^7^ For comparison, the survival was almost 3 years in a study involving a population of patients who were selected regarding medical factors (eg, Gleason score, prostate-specific antigen (PSA), metastasis site), and who had mild symptoms and a good performance status (0–1).^8^

To ensure the best possible quality of life (QoL) in patients with incurable prostate cancer, symptom management is important throughout the disease, but even more important is finding the balance between enhancing QoL and prolonging life. It has been shown that men treated for mCRPC hope that the treatment will prolong life and relieve symptoms, while allowing them good QoL during the remainder of their life.^9^ They also actively weigh potential treatment benefits against possible treatment side effects and worsening QoL.^9^ Seeking such balance is also in line with recommendations for an early integration of a palliative care approach in conjunction with life-prolonging treatments for incurable illness.^10 11^

Few recent studies have been conducted regarding changes in symptoms in men during treatment for mCRPC, and to our knowledge, none with a multidimensional approach. In a mixed-methods study, it was shown that symptom management is not optimal during treatment for mCRPC.^12^ When starting treatment, patients reported pain as the worst symptom, whereas after 3 months of systemic therapy, fatigue was the worst.^12^ In a study with a real-world approach regarding health-related QoL (HRQoL), pain severity was shown to be fairly low in the early phase of mCRPC, but increased over time, along with deterioration in the role and physical domains of HRQoL.^13^

Despite the expanding treatment field of mCRPC and the importance of symptom management for enhancing QoL, there are few studies about men’s experiences of symptom burden over time when receiving one or more lines of treatment in a real-world situation.^12^,^14^ Hence, the aim of this study was to investigate changes in symptom burden during the first year of life-prolonging treatment of mCRPC.

## Methods

### Design and sample

This study was part of a longitudinal, prospective multicentre project^9 15^ (PROCEED) that follows men with mCRPC during life-prolonging treatment in a real-world context. The Strengthening the Reporting of Observational Studies in Epidemiology guidelines were used when reporting the study.^16^

Due to the real-world approach, the inclusion criteria for the project were few: those who were able to understand Swedish and those who were about to start their first line of treatment for mCRPC. The participants were recruited between April 2015 and March 2022 at four oncology departments in Sweden, both university and county hospitals. A priori power analysis was conducted for the overall project based on clinically relevant changes in one of the instruments (not used in this study),^17^ showing that a sample of 120–150 men would be sufficient. Given that the severity of the patients’ illness increased the likelihood of dropouts due to health decline or death, the goal was set to include 150 men. Oral and written information were given to eligible men. All participants provided written informed consent in accordance with the Declaration of Helsinki.^18^

### Data collection

**Figure 1:**
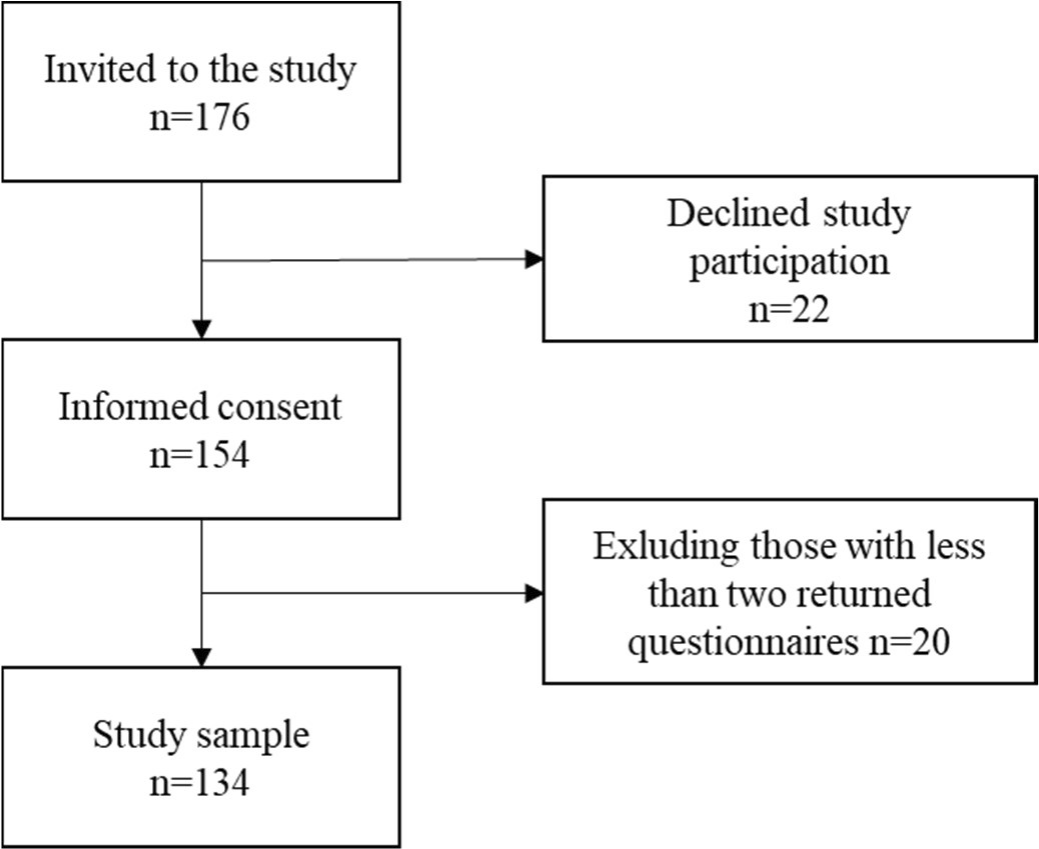
Study enrolment flow chart.

Data were collected through repeated questionnaires sent out by mail approximately every third month, and in case of changed treatment, during a 2-year period. Clinical data, including PSA values and analgesic use, were collected from the medical record at inclusion and in conjunction with every follow-up questionnaire. Medical data were monitored by an independent monitor for the purpose of quality assurance. In the present study, data from the first year of inclusion were used. If a questionnaire was not returned within 2–4 weeks, up to two reminders were sent. A total of 176 men were invited to participate and 154 men gave informed consent. Men who had completed at least two questionnaires, regardless of time points, were included in the sample for this study (figure 1). Data from five time points (t1–t5) during the first year after inclusion were used.

### Measures

Symptoms were measured using the self-administered Memorial Symptom Assessment Scale (MSAS),^19^ chosen based on its multidimensional approach. The original MSAS assesses 32 physical and psychological symptoms. For 24 of these 32 symptoms, 3 dimensions—frequency, severity and distress—are rated. The other eight symptoms are rated only for the severity and distress dimensions. Higher scores indicate greater frequency, severity and distress.^20 21^ The MSAS question about problems with sexual activity and interest was split into two questions, motivated by the fact that all the men in this study were surgically or medically castrated and the activity or desire may be different from that of a non-castrated group. Thus, the MSAS in the present study included 33 questions.

The MSAS consists of three subscales and in this study, three measures of symptom burden from MSAS were used: the MSAS physical (MSAS-PHYS) subscale score (1–4), the MSAS psychological (MSAS-PSYCH) subscale score (1–4) and the number of symptoms, based on the reported occurrence of symptoms (Did you have any of the following symptoms? No/yes) (0–33 symptoms). MSAS-PHYS consists of 12 physical symptoms (lack of appetite, lack of energy, pain, feeling drowsy, constipation, dry mouth, nausea, vomiting, change in taste, weight loss, feeling bloated and dizziness). MSAS-PSYCH consists of six psychological symptoms (feeling sad, worrying, feeling irritable, feeling nervous, difficulty sleeping and difficulty concentrating). The subscales are calculated according to Portenoy *et al*^19^; in brief, the dimension scores (for frequency, severity and distress) are summed and then divided by the number of dimensions for which the patient gave an answer.

Demographic variables included were age, marital status (married-cohabiting/single-widowed) and educational level (elementary school/high school/university). Additional data were collected from the medical records at inclusion, such as years since primary diagnosis, years since diagnosis of metastatic disease, tumour classification (TNM and Gleason score), PSA, haemoglobin, alkaline phosphatase and analgesic use (yes/no). From the medical record, also the type(s) of life-prolonging treatment(s) were collected.

### Data analysis

Descriptive statistics were used to present background characteristics and medical data about the included men. Continuous variables are described with median and IQR, mean and SD, and categorical data as proportions and percentages. Symptoms reported by 50% or more of the men at any time point are described regarding frequency, severity and distress for each time point. Comparisons between clinical data at inclusion between those who did not complete the 1-year period and those who completed were conducted using the Mann-Whitney U-test and χ^2^ test for continuous and categorical data, respectively.

Linear mixed models (LMMs) for repeated measures were used to analyse changes in symptom burden over time after adjusting for possible covariates. Three separate models were used to assess the three symptom burden outcomes from the MSAS questionnaire, namely physical symptoms (MSAS-PHYS), psychological symptoms (MSAS-PSYCH) and the number of symptoms. The correlations between the included variables were all below 0.70. Since the time span between the data collection time points was uneven, an unstructured covariance matrix was chosen. The LMM was adjusted for covariates measured at inclusion (age, education, marital status, years since metastatic disease and analgesic use) as fixed effects. A backwards LMM was performed and AIC (Akaike information criterion) was used to find the best model. No imputation of data was considered necessary as the LMMs provide estimates using all available data. The significance level was set to ≤0.05. All statistical analyses were performed by using IBM SPSS V.27 (IBM).

## Results

### Sample and characteristics

From the sample of 154 men, 134 returned at least 2 questionnaires during the 1-year follow-up and were thus included in the analyses (figure 1). In total, data from 576 questionnaires were analysed, together with medical data. The average age of the included men was 75 years (range 50–88) at the time of inclusion and a median of 3 years had passed since their primary prostate cancer diagnosis (range 0–22) (table 1). At the time of diagnosis, 44.8% of the men already had metastases (M1). At inclusion in the study, 89 (66.4%) of the men had bone metastases while 37 (27.6%) had metastases in lymph nodes, 3 (2.2%) in the lungs and 2 (1.5%) in the liver. In most cases, the first line of treatment was a second-generation antiandrogen (enzalutamide or abiraterone).

**Table 1:** Sociodemographic and medical characteristics of 134 men with metastatic castration-resistant prostate cancer starting life-prolonging treatment: frequencies, percentages, mean, SD, median, IQR, minimum-maximum (min-max)

Age (years)*	Mean (SD)	75.2 (7.0)	
	Median (IQR)	75.0 (9.2)	
	Min-max	50.0–88.0	
	Missing	–	
Years since primary diagnosis	Mean (SD)	4.6 (4.8)	
	Median (IQR)	3.0 (5.2)	
	Min-max	0.0–22.0	
	Missing	4	
Years since diagnosis of metastatic disease	Mean (SD)	1.3 (2.0)	
	Median (IQR)	1.0 (2.0)	
	Min-max	0.0–13.0	
	Missing	1	
PSA (ng/mL)*	Mean (SD)	68.8 (125.6)	
	Median (IQR)	29.5 (68.5)	
	Min max	0.5–1141.0	
	Missing	–	
Haemoglobin (g/L)*	Mean (SD)	132.2 (13.3)	
	Median (IQR)	132.5 (16.2)	
	Min-max	89.0–171.0	
	Missing	–	
Alkaline phosphatase (µkat/L)*	Mean (SD)	3.0 (4.3)	
	Median (IQR)	1.6 (1.6)	
	Min-max	0.6–28.0	
	Missing	–	
		n	%
Marital status	Married/cohabiting	96	71.6
	Single/widowed	31	23.1
	Other	1	0.7
	Missing	6	4.5
Education	Elementary school	57	42.5
	High school	40	29.9
	University	33	24.6
	Missing	4	2.9
Tumour (T) stage†	T1	11	8.2
	T2	27	20.1
	T3	66	49.3
	T4	20	14.9
	Tx	5	3.7
	Missing	5	3.7
Node (N) stage†	N0	75	56.0
	N1	41	30.6
	Nx	13	9.7
	Missing	5	3.7
Metastasis (M) stage†	M0	70	53.4
	M1	60	44.8
	Mx	1	0.7
	Missing	4	3.0
Gleason score†	6	13	9.7
	7	43	32.1
	8	34	25.4
	9	31	23.1
	10	2	1.5
	Missing	11	8.2
Metastasis site*	Bone	89	66.4
	Lymph nodes	37	27.6
	Lung	3	2.2
	Liver	2	1.5
	Other	1	0.7
	Missing	2	1.5
Treatment	Abiraterone	17	12.6
	Docetaxel	38	28.4
	Enzalutamide	69	51.5
	Radium-223	4	3.0
	Cabazitaxel	3	2.3
	Other‡	3	2.3
	Missing	–	
Analgesic use*	Yes/no	66/65	50.4/49.6
	Missing	–	

* At inclusion.

† At primary diagnosis.

‡ Abiraterone or enzalutamide plus study drug/placebo.

PSAprostate-specific antigen

Of the men included, 43 (32.1%) changed treatment during follow-up. Of those who changed treatment, eight (6.0%) had three lines of treatment. Of the 134 men included in the analyses, 14 (10.4%) died during the follow-up, 8 (6.0%) withdrew their consent and 3 (2.2%) failed to follow-up. There were significant differences regarding PSA values (U=890.0, p=0.008) and time since primary diagnosis (U=865.5, p=0.005) between these 25 men (PSA, median=60.0, time since primary diagnosis, median=1.0 year) and the 108 men who completed the 1-year period (PSA=26.0, time since primary diagnosis, median=4.0 years). A significant difference was also found between the two groups regarding N stage (χ^2^=11.4, p=0.022).

#### Description of common symptoms over time

**Figure 2:**
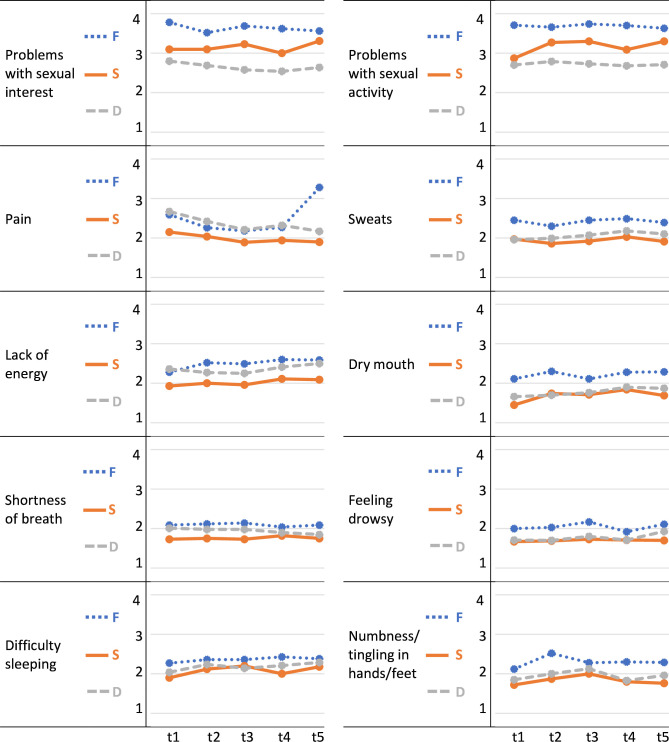
Frequency (F), severity (S) and distress (D) for symptoms reported by ≥50% of the men at any of the time points t1–t5.

10 symptoms were reported by at least 50% of the men at different time points. These were lack of energy, sweats, problems with sexual activity, problems with sexual interest, pain, dry mouth, shortness of breath, feeling drowsy, numbness/tingling in hands/feet and difficulty sleeping (figure 2). Sweats and lack of energy alternated as the most reported symptom at the different time points. The two aspects of sexual problems were the symptoms with the highest scores in all dimensions, although the distress scores were lower than both the frequency and the severity scores. Pain was the only symptom that had higher or equal distress scores than frequency and severity scores, except for the last time point (t5) where frequency peaks.

#### Symptom burden

On average, the men had 10.25 (SD 7.30, min-max 0–31) symptoms at inclusion (t1) and at the last time point (t5) they had a mean of 12.14 (SD 7.93, min-max 0–33) symptoms (table 2). The MSAS-PHYS and MSAS-PSYCH subscales had higher scores at t5 compared with t1, indicating a higher symptom burden.

**Table 2:** Symptom burden of 134 men with metastatic castration-resistant prostate cancer, who completed at least two questionnaires at any time point: mean, SD, median, IQR, min-max.

	t1 (n=134)	t2 (n=134)	t3 (n=119)	t4 (n=109)	t5 (n=80)
Number of symptoms					
Mean (SD)	10.25 (7.30)	11.92 (7.73)	12.08 (7.75)	11.50 (6.92)	12.14 (7.93)
Median (IQR)	9.00 (11.25)	11.00 (9.00)	11.00 (10.00)	11.00 (10.00)	11.00 (12.00)
Min-max	0–31	0–33	1–33	1–33	0–33
Missing	–	–	–	1	2
Physical symptoms (MSAS-PHYS subscale)					
Mean (SD)	0.47 (0.48)	0.64 (0.56)	0.57 (0.52)	0.58 (0.53)	0.63 (0.65)
Median (IQR)	0.35 (0.80)	0.50 (0.78)	0.46 (0.75)	0.49 (0.71)	0.47 (0.94)
Min-max	0.00–2.06	0.00–2.62	0.00–2.10	0.00–2.38	0.00–3.04
Missing	–	–	–	–	–
Psychological symptoms (MSAS-PSYCH subscale)					
Mean (SD)	0.44 (0.56)	0.51 (0.67)	0.49 (0.66)	0.47 (0.62)	0.59 (0.75)
Median (IQR)	0.20 (0.77)	0.16 (0.92)	0.20 (0.81)	0.25 (0.75)	0.24 (0.96)
Min-max	0.00–2.50	0.00–2.50	0.00–3.06	0.00–2.34	0.00–3.00
Missing	–	–	–	–	–

MSASMemorial Symptom Assessment Scale

The LMM test of fixed effects of changes in physical symptom burden over time showed a significant increase from t1 to t5 (estimate=0.16, p=0.00) (online supplemental table 1). Having a low education level (elementary school) was significantly associated with the change in physical symptom burden (estimate=−0.25, p=<0.01) as was the use of analgesics at t1 (estimate=−0.16, p=0.04). The psychological symptom burden did not change significantly over time (estimate=0.10, p=0.10). The number of symptoms changed significantly from t1 to t5 (estimate=1.87, p=0.02).

## Discussion

To our knowledge, this is the first real-world longitudinal multidimensional study of symptom burden among men receiving one or more lines of treatment for mCRPC. The aim was to investigate changes in symptom burden during the first year of life-prolonging treatment. Previous research has focused on HRQoL along with descriptions of some symptoms, often pain.^13 14^ The results may give valuable information useful for symptom management in enhancing the best possible QoL.

Of the 10 symptoms reported by 50% or more of the men, 9 were physical. The two aspects of sexual problems were the symptoms with the highest frequency and severity scores at all time points, which is not surprising given the castration therapy all men had. That men in this phase report frequent sexual problems has been shown before.^22^ In all stages of the disease, sexual problems are frequently reported and highly prioritised by the men. But in the CRPC-phase, it is scored lower than pain, fatigue, physical activity and urinary frequency problems.^23^ It is possible that the sexual problems and the distress they may cause often are overlooked in the clinical situation in this late phase of the disease and in this age group. Old age in combination with having a life-limiting disease may influence how these symptoms are met by healthcare professionals. Hence, these problems need to be more acknowledged by healthcare professionals to relieve distress although the underlying causes may be difficult to solve.

Sweats and lack of energy were the most commonly reported symptoms. Sweats are probably related to the castration therapy and although this was a frequently reported symptom; the severity and distress rates were not very high and were quite stable over time. Sweats alternated with lack of energy as the symptom most often reported as occurring at the different time points. Lack of energy was also fairly stable, with a small increase, in all dimensions during the 1-year follow-up. The MSAS questionnaire uses the concept ‘lack of energy’ which may be similar to the concept ‘fatigue’ that is more often used in this context. Fatigue has been shown to be the symptom most frequently reported by men with mCRPC, followed by pain.^22 24^ Furthermore, fatigue has been shown to affect QoL,^25^ as well as being one of the health problems most highly prioritised by this group, surpassed only by pain.^23^

Pain is a common symptom in men with mCRPC,^13 14 24^ and even if it was not reported as the most common symptom in the present study, it was one of the ten most reported symptoms. Pain was also the only symptom for which the distress scores were equal or higher than the frequency and severity scores at all time points except the last one (t5). That almost 50% of the men reported pain at t1 may indicate that pain management was not sufficient even if the symptom seemed to be recognised and 50% of the men were using analgesic drugs already at inclusion. The pain these men are suffering from may be related to bone metastases^26^ and one aim with treatment in this phase is also symptom reduction why the treatment itself may be part of the symptom management. However, the different dimensions of pain were rather stable over time even if the frequency where at the highest at the last time point. This indicates that it would be interesting to follow the development of pain after this first year.

The physical symptom subscale, as a measure of symptom burden, was the only one that changed significantly during the follow-up. The mean score was relatively low at t1 (0.47) but was higher (worse physical symptom burden) at t5 (0.63), and highest at t2 (0.64). This is the opposite of patients with newly diagnosed cancer at stages II–III where the scores decreased over a 1-year period^27^ The high value at t2 may be related to that 30% of the men were receiving chemotherapy with accompanying symptoms/side effects. The corresponding value for patients with colorectal cancer during their second or third cycle of chemotherapy is 0.58.^28^ The increase in symptom scores for the men with mCRPC is probably related to their progressive disease, where the frequency, severity and distress caused by each symptom may increase, together with that new symptoms may appear.

The only psychological symptom among the top 10 reported during the 5 time points was difficulty sleeping. Questions can be raised about why the men did not report higher levels of psychological symptoms since psychological distress has been shown to be related to advanced cancer and to progressive disease.^15 29 30^ In a study comparing groups with low and high number of symptoms using MSAS (0–12 symptoms vs 13–32 symptoms), Gilbertson-White *et al*^31^ found that there were more psychological symptoms among the top 12 symptoms in the group of those with many symptoms (13–32 symptoms) than in the group with fewer symptoms (0–12 symptoms). By this definition, in the present study, many men are in the low symptom group even if the average number of symptoms is just above 12 at the last time point (t5).

Psychological symptom burden did not change significantly over time. This was quite surprising given that several men had a progression during the follow-up. Distress has been described in terms of fear of progression by men with advanced prostate cancer, who also describe uncertainty about the time they may have ahead.^32^ Physical symptoms together with progression may also induce fears about functional decline, about being dependent, and also about death and dying.^15^

The range in number of symptoms was large: some men reported up to 33 symptoms while others did not report any symptoms at all, nevertheless there was a significant change over time. The presence of many symptoms may be a sign that life is approaching the end^33^ and should be taken into consideration by the healthcare professionals interacting with these men. The high number of symptoms reported by some men may also indicate a need for improved symptom management.

Management of symptoms that men with mCRPC are experiencing is of great importance and is in line with the recommendation of an early integrated palliative care approach aiming to ensure best possible QoL. The International Association for Hospice and Palliative Care stresses that in order to improve or retain QoL, early identification and assessment of physical, psychosocial and spiritual symptoms is needed.^10^ In this group of men with incurable disease, it is, therefore, relevant to adopt a palliative approach during the whole trajectory from starting life-prolonging treatment to the end of life, including adequate symptom management. If problems are managed early, unnecessary suffering can be avoided. The findings from the present study indicate that there are unmet needs regarding management of sexual problems and other physical symptoms, such as pain and lack of energy, among others. A thorough symptom assessment may help find the individuals with the greatest need for support to deal with both physical and psychological symptoms. The professional team at the oncology department should be able to manage most of the palliative care needs of this group of patients, and for the more difficult cases they can request help from the specialist palliative care team.^10 34 35^

### Strengths and limitations

A strength of this study is the longitudinal design, and that a large proportion of the men agreed to participate. Given that this is a group of seriously ill patients, the large proportion of men who completed all follow-ups must also be considered a strength. The attrition rate was 18.6% whereas in a global study among patients with advanced cancer the attrition rate was 33%.^36^ The mean age was somewhat higher than in other studies of the population of men with mCRPC.^14 37^ This may be related partly to that the ‘real-world’ sampling used in this study was successful in including patients that would not be considered for some treatment studies. However, the old age in combination with the fact that most of the men had a second-generation antiandrogen (enzalutamide or abiraterone) as the first line of treatment may also indicate a limitation due to selection bias. Some men may have been lost to treatment studies including chemotherapy. Another strength of this study is that we used a well-validated and multidimensional measure of symptoms and symptom burden.^19 20^ Furthermore, a strength is that medical data were collected from the medical records not only concerning primary treatment and disease classification data at primary diagnosis but also concerning actual treatment and medical follow-up.

## Conclusion

Understanding the different dimensions of symptoms, particularly physical symptoms and changes in symptom burden, of men with mCRPC during the first year of life-prolonging treatment may contribute to effective symptom management. Early integration of a palliative care approach may be helpful in order to ensure the best possible QoL in this late phase of the disease. To develop a full picture of the symptom burden of men with mCRPC, additional studies with a longer follow-up period may be beneficial. Also, qualitative studies may give a deeper understanding of the men’s experiences of the overall symptom burden, as well as the meaning of the different dimensions of the symptom experiences.

## supplementary material

10.1136/spcare-2024-005054 online supplemental table 1

## Data Availability

The datasets generated and/or analysed during the current study are not publicly available due to Swedish law and data regulations. For questions about data that support the findings of this study, contact AW-L.

## References

[1] Sung H, Ferlay J, Siegel RL (2021). Global cancer statistics 2020: GLOBOCAN estimates of incidence and mortality Worldwide for 36 cancers in 185 countries. CA Cancer J Clin.

[2] Beer TM, Armstrong AJ, Rathkopf DE (2014). Enzalutamide in metastatic prostate cancer before chemotherapy. N Engl J Med.

[3] Ryan CJ, Smith MR, de Beno JS (2013). Abiraterone in metastatic prostate cancer without previous chemotherapy. N Engl J Med.

[4] Tannock IF, de Wit R, Berry WR (2004). Docetaxel plus prednisone or mitoxantrone plus prednisone for advanced prostate cancer. N Engl J Med.

[5] de Bono JS, Oudard S, Ozguroglu M (2010). Prednisone plus cabazitaxel or mitoxantrone for metastatic castration-resistant prostate cancer progressing after docetaxel treatment: a randomised open-label trial. Lancet.

[6] Hoskin P, Sartor O, O’Sullivan JM (2014). Efficacy and safety of radium-223 dichloride in patients with castration-resistant prostate cancer and symptomatic bone metastases, with or without previous docetaxel use: a prespecified subgroup analysis from the randomised, double-blind, phase 3 ALSYMPCA trial. Lancet Oncol.

[7] Aly M, Leval A, Schain F (2020). Survival in patients diagnosed with castration-resistant prostate cancer: a population-based observational study in Sweden. Scand J Urol.

[8] Armstrong AJ, Lin P, Tombal B (2020). Five-year survival prediction and safety outcomes with enzalutamide in men with chemotherapy-naïve metastatic castration-resistant prostate cancer from the PREVAIL trial. Eur Urol.

[9] Doveson S, Holm M, Axelsson L (2020). Facing life-prolonging treatment: the perspectives of men with advanced metastatic prostate cancer - An interview study. Eur J Oncol Nurs.

[10] Radbruch L, De Lima L, Knaul F (2020). Redefining palliative care-A new consensus-based definition. J Pain Symptom Manage.

[11] Ferrell BR, Temel JS, Temin S (2017). Integration of palliative care into standard oncology care: American society of clinical oncology clinical practice guideline update. J Clin Oncol.

[12] Catt S, Matthews L, May S (2019). Patients’ and partners’ views of care and treatment provided for metastatic castrate-resistant prostate cancer in the UK. Eur J Cancer Care (Engl).

[13] Kuppen MCP, Westgeest HM, van den Eertwegh AJM (2020). Health-related quality of life and pain in a real-world castration-resistant prostate cancer population: results from the PRO-CAPRI study in the Netherlands. Clin Genitourin Cancer.

[14] Jenkins V, Solis-Trapala I, Payne H (2019). Treatment experiences, information needs, pain and quality of life in men with metastatic castrate-resistant prostate cancer: results from the EXTREQOL study. Clin Oncol.

[15] Rönningås U, Holm M, Doveson S (2022). Signs and symptoms in relation to progression, experiences of an uncertain illness situation in men with metastatic castration-resistant prostate cancer-A qualitative study. Eur J Cancer Care (Engl).

[16] von Elm E, Altman DG, Egger M (2007). The strengthening the reporting of observational studies in epidemiology (STROBE) statement: guidelines for reporting observational studies. Lancet.

[17] Cella D, Nichol MB, Eton D (2009). Estimating clinically meaningful changes for the functional assessment of cancer therapy--Prostate: results from a clinical trial of patients with metastatic hormone-refractory prostate cancer. Value Health.

[18] World Medical Association (2013). World Medical Association Declaration of Helsinki: ethical principles for medical research involving human subjects. JAMA.

[19] Portenoy RK, Thaler HT, Kornblith AB (1994). The memorial symptom assessment scale: an instrument for the evaluation of symptom prevalence, characteristics and distress. Eur J Cancer.

[20] Browall M, Kenne Sarenmalm E, Nasic S (2013). Validity and reliability of the Swedish version of the Memorial Symptom Assessment Scale (MSAS): an instrument for the evaluation of symptom prevalence, characteristics, and distress. J Pain Symptom Manage.

[21] Chang VT, Hwang SS, Thaler HT (2004). Memorial symptom assessment scale. Expert Rev Pharmacoecon Outcomes Res.

[22] Holmstrom S, Naidoo S, Turnbull J (2019). Symptoms and Impacts in metastatic castration-Resistant prostate cancer: qualitative findings from patient and physician interviews. Pat.

[23] Bryant-Lukosius D, Browne G, DiCenso A (2010). Evaluating health-related quality of life and priority health problems in patients with prostate cancer: a strategy for defining the role of the advanced practice nurse. CONJ.

[24] Drudge-Coates L, Oh WK, Tombal B (2018). Recognizing symptom burden in advanced prostate cancer: a global patient and caregiver survey. Clin Genitourin Cancer.

[25] Rodríguez Antolín A, Martínez-Piñeiro L, Jiménez Romero ME (2019). Prevalence of fatigue and impact on quality of life in castration-resistant prostate cancer patients: the VITAL study. BMC Urol.

[26] Gater A, Abetz-Webb L, Battersby C (2011). Pain in castration-resistant prostate cancer with bone metastases: a qualitative study. Health Qual Life Outcomes.

[27] Deshields TL, Potter P, Olsen S (2014). The persistence of symptom burden: symptom experience and quality of life of cancer patients across one year. Support Care Cancer.

[28] Pettersson G, Berterö C, Unosson M (2014). Symptom prevalence, frequency, severity, and distress during chemotherapy for patients with colorectal cancer. *Support Care Cancer*.

[29] An E, Wennberg E, Nissim R (2020). Death talk and relief of death-related distress in patients with advanced cancer. BMJ Support Palliat Care.

[30] Levy A, Cartwright T (2015). Men’s strategies for preserving emotional well-being in advanced prostate cancer: an interpretative phenomenological analysis. Psychol Health.

[31] Gilbertson-White S, Aouizerat BE, Jahan T (2012). Determination of cutpoints for low and high number of symptoms in patients with advanced cancer. J Palliat Med.

[32] Chambers SK, Hyde MK, Laurie K (2018). Experiences of Australian men diagnosed with advanced prostate cancer: a qualitative study. BMJ Open.

[33] Holm M, Doveson S, Lindqvist O (2018). Quality of life in men with metastatic prostate cancer in their final years before death - a retrospective analysis of prospective data. BMC Palliat Care.

[34] Swami M, Case AA (2018). Effective palliative care: what is involved?. Oncol (Willist Park).

[35] Touzel M, Shadd J (2018). Content validity of a conceptual model of a palliative approach. J Palliat Med.

[36] Perez-Cruz PE, Shamieh O, Paiva CE (2018). Factors associated with attrition in a multicenter longitudinal observational study of patients with advanced cancer. J Pain Symptom Manage.

[37] Andrews JR, Ahmed ME, Karnes RJ (2020). Systemic treatment for metastatic castrate resistant prostate cancer: does seqence matter?. Prostate.

