# Supplementary material for: Symptom burden among men treated for castration-resistant prostate cancer: a longitudinal study
Source: BMJ Support Palliat Care. 2024 Aug 8;15(1):e005054. doi: 10.1136/spcare-2024-005054 (PMC11874351; doi:10.1136/spcare-2024-005054)
Supplement: online supplemental table 1 [file spcare-15-1-s001.docx]

Supplemental table 1. Change in symptom burden (physical symptoms, Model 1; psychological symptoms, Model 2; number of symptoms, Model 3) over one year adjusted for covariates (measured at inclusion) (*n*=126)

|  | **Model 1, physical symptoms** | | | | **Model 2, psychological symptoms** | | | | **Model 3, number of symptoms** | | | |
| --- | --- | --- | --- | --- | --- | --- | --- | --- | --- | --- | --- | --- |
| Variable | Estimate (95% CI) | p | Estimate (95% CI) | p | Estimate (95% CI) | p | Estimate (95% CI) | p | Estimate (95% CI) | p | Estimate (95% CI) | p |
| Intercept | 0.66 (-0.17, 1.49) | 0.12 | 0.67 (0.50, 0.84) | 0.00 | 0.93 (-0.11, 1.98) | 0.08 | 0.53 (0.32, 0.75) | 0.00 | 15.93 (3.66, 28.19) | 0.01 | 10.78 (8.17, 13.40) | 0.00 |
| t1 | 0^b^ |  | 0^b^ |  | 0^b^ |  | 0^b^ |  | 0^b^ |  | 0^b^ |  |
| **t2** | 0.15 (0.06, 0.25) | 0.00 | **0.15 (0.06, 0.25)** | **0.00** | 0.07 (-0.01, 0.16) | 0.94 | 0.07 (-0.01, 0.16) | 0.08 | 1.37 (0.05, 2.68) | 0.04 | **1.40 (0.09, 2.71)** | **0.04** |
| **t3** | 0.12 (0.03, 0.22) | 0.01 | **0.12 (0.03, 0.22)** | **0.01** | 0.04 (-0.07, 0.15) | 0.47 | 0.04 (-0.07, 0.14) | 0.46 | 2.00 (0.55, 3.46) | 0.01 | **2.04 (0.60, 3.48)** | **0.01** |
| **t4** | 0.12 (0.01, 0.22) | 0.03 | **0.12 (0.01, 0.22)** | **0.02** | 0.02 (-0.08, 0.13) | 0.62 | 0.03 (-0.08, 0.13) | 0.60 | 1.35 (-0.06, 2.77) | 0.61 | 1.36 (-0.05, 2.76) | 0.06 |
| **t5** | 0.16 (0.05, 0.27) | 0.00 | **0.16 (0.05, 0.26)** | **0.00** | 0.10 (-0.02, 0.22) | 0.09 | 0.10 (-0.02, 0.22) | 0.10 | 1.87 (0.24, 3.50) | 0.02 | **1.87 (0.25, 3.50)** | **0.02** |
| University | 0^b^ |  | 0^b^ |  | 0^b^ |  | 0^b^ |  | 0^b^ |  | 0^b^ |  |
| High School | 0.02 (-0.18, 0.22) | 0.86 | 0.01 (-0.19, 0.20) | 0.96 | 0.02 (-0.22, 0.27) | 0.84 | 0.02 (-0.23, 0.27) | 0.86 | -0.43 (-3.36, 2.50) | 0.77 | -0.67 (-3.63, 2.30) | 0.66 |
| **Elementary school** | -0.24 (-0.42, -0.06) | 0.01 | **-0.25 (-0.43, -0.07)** | **0.01** | -0.04 (-0.27, 0.19) | 0.71 | -0.08 (-0.30, 0.15) | 0.51 | 0.53 (-2.17, 3.24) | 0.70 | 0.64 (-2.08, 3.36) | 0.64 |
| **Analgesic use^1^** | -0.16 (-0.31, -0.01) | 0.03 | **-0.16 (-0.30, -0.01)** | **0.04** | -0.14 (-0.33, 0.04) | 0.14 | -0.10 (-0.29, 0.08) | 0.27 | -0.40 (-2.62, 1.82) | 0.72 | -0.46 (-2.68, 1.77) | 0.69 |
| Marital status^2^ | 0.03 (-0.15, 0.20) | 0.76 | - | - | 0.18 (-0.04, 0.40) | 0.11 | - | - | 0.61 (-1.94, 3.17) | 0.64 | - | - |
| Time since metastatic disease | -0.01 (-0.05, 0.02) | 0.41 | - | - | -0.00 (-0.05, 0.04) | 0.96 | - | - | -0.35 (-0.90, 0.20) | 0.21 | - | - |
| Age | 0.00 (-0.01, 0.01) | 0.99 | - | - | -0.01 (-0.02, 0.01) | 0.31 | - | - | -0.07 (-0.23, 0.09) | 0.38 | - | - |

^1^Analgesic use, no as reference

^2^Marital status, married/cohabiting as reference
